# Subchondral insufficiency fractures, subchondral insufficiency fractures with osteonecrosis, and other apparently spontaneous subchondral bone lesions of the knee—pathogenesis and diagnosis at imaging

**DOI:** 10.1186/s13244-023-01495-6

**Published:** 2023-10-02

**Authors:** Jacques Malghem, Frédéric Lecouvet, Bruno Vande Berg, Thomas Kirchgesner, Patrick Omoumi

**Affiliations:** 1 Department of Radiology, Cliniques Universitaires Saint-Luc, UCLouvain, Avenue Hippocrate 10, 1200 Brussels, Belgium; 2 Department of Medical Imaging, Clinique CHC Montlégia, Boulevard Patience Et Beaujonc 2, 4000 Liège, Belgium; 3 Department of Diagnostic and Interventional Radiology, Lausanne University Hospital (CHUV) and University of Lausanne, Rue du Bugnon 46, 1010 Lausanne, Switzerland

**Keywords:** Insufficiency fracture, Spontaneous osteonecrosis of the knee, Bone marrow edema, Knee, Subchondral bone

## Abstract

Subchondral insufficiency fractures (SIFs) and SIFs with osteonecrosis (SIF-ONs) of the knee (previously misnamed spontaneous osteonecrosis of the knee (SONK)) are bone lesions that appear without prior traumatic, tumoral, or inflammatory event.

Both conditions are characterized in the early stages by epiphyseal bone marrow edema (BME)-like signal at MRI. However, while SIFs usually heal spontaneously, they can also evolve to osteonecrosis (i.e., SIF-ON), which may progress to an irreversible collapse of the articular surface. Careful analysis of other MRI signs may help differentiate the two conditions in the early phase. In SIFs, the BME edema-like signal extends to the area immediately adjacent to the subchondral plate, while in SIF-ONs, this subchondral area shows low signal intensity on fluid-sensitive MR images due to altered bone marrow. The thickness and length of subchondral areas with low fluid-sensitive signal intensity are important factors that determine the prognosis of SIF-ONs. If they are thicker than 4 mm or longer than 14 mm, the prognosis is poor. The differential diagnosis of SIFs and SIF-ONs include bone lesions associated with the “complex regional pain syndrome” (CRPS), epiphyseal osteonecrosis of systemic origin, and those related to cartilage pathology.

**Clinical relevance statement **Imaging plays an essential role in diagnosing subchondral insufficiency fractures (SIFs) from subchondral insufficiency fractures with osteonecrosis (SIF-ONs) and collapse, as well as in distinguishing them from other spontaneous knee subchondral bone lesions presenting with bone marrow edema-like signal.

**Key points**

• Subchondral insufficiency fractures may affect the knee, especially in older adults.

• Subchondral insufficiency fractures usually heal spontaneously.

• Sometimes, subchondral osteonecrosis and collapse may complicate subchondral insufficiency fractures.

• Bone marrow-like edema is an aspecific sign seen in all these lesions.

• Degraded marrow in osteonecrosis complicating fractures is hypointense on fluid-sensitive sequences.

**Graphical Abstract:**

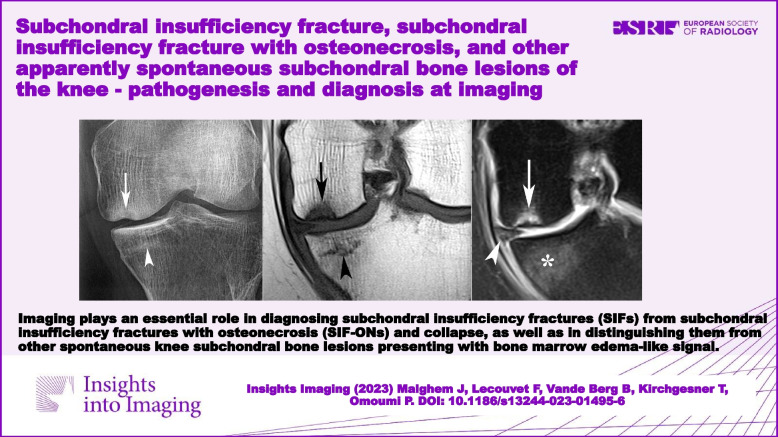

## Background

This manuscript deals with various lesions affecting the knee epiphyses; occurring in the absence of prior trauma, tumor, inflammatory, or infectious disease; and mainly presenting as bone marrow edema (BME)-like signal at MRI, or as an epiphyseal collapse.

The lesions associated with BME-like signal changes can either heal spontaneously or progress to epiphyseal collapse associated with subchondral osteonecrosis. So, it is important to differentiate between the underlying conditions associated with BME-like signal. However, this can be a difficult task due to many sources of confusion.

The first source of difficulty is related to the fact the BME-like signal is an aspecific MRI sign which may be associated with very different pathophysiological processes. For example, BME-like signal can be the consequence of insufficiency fractures or associated with a clinical entity that is not fully understood, called “complex regional pain syndrome” (CRPS) (previously referred to as “reflex sympathetic dystrophy syndrome, algodystrophy, or transient osteoporosis”). In addition, similar lesions can be observed in osteoarthritis, which may be associated with a variable amount of subchondral BME-like signal and osteonecrosis.

Second, some of the conditions associated with BME-like signal may themselves have very different origins. Epiphyseal osteonecrosis may for example complicate insufficiency fractures, but it can also be of ischemic origin, due to various systemic hemodynamic or metabolic disorders.

Third, there is great confusion around the terminology used in the literature. For example, some authors use the term “secondary osteonecrosis” in reference to osteonecrosis complicating insufficiency fractures [1], while others use it to designate osteonecrosis of systemic origin [2, 3]. Another source of confusion is the use of the term “Spontaneous osteonecrosis of the knee” (SONK) to either refer to spontaneous lesions that can heal or to those that are complicated by collapse [4, 5]. Others have used using the term “Subchondral insufficiency fracture” (SIF) to refer to both types of lesions: those which heal spontaneously and those that progress to collapse [6–8]. This is reflected in a recent opinion paper where the experts of the “International Skeletal Society” have proposed to completely abandon the term “SONK” in favor of “SIF” [1, 9].

In this review, we discuss the pathological conditions associated with BME-like signal in the knee epiphysis, presenting key imaging features allowing the differential diagnosis between lesions that may have a very different prognosis.

**Fig. 1:**
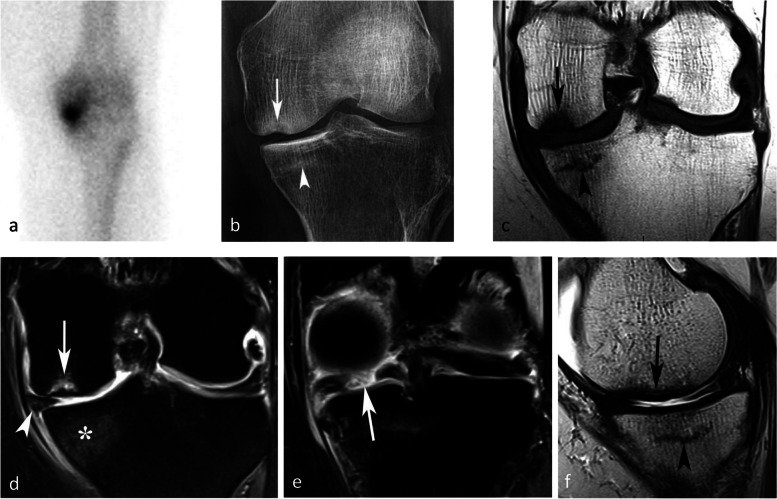
Subchondral insufficiency fracture (SIF) and subchondral insufficiency fracture complicated with osteonecrosis (SIF-ON). **a** Bone scan in a woman who presented with sudden onset of pain for several weeks, showing intense uptake in the medial femorotibial compartment. **b** Two months later, radiograph shows focal collapse of the inferior pole of the medial condyle (arrow) with sclerosis of the adjacent cancellous bone, and a sclerotic horizontal band parallel to the medial tibial plateau (arrowhead). **c** MR shows a focal very low signal intensity on coronal T1-weighted image in the medial condyle lower pole (arrow) and a thin low signal intensity horizontal band under the medial tibial plateau (arrowhead). Fat-suppressed T2-weighted image (**d**), showing heterogeneous signal in the condylar lesion (arrow) and a bone marrow moderate high signal under the tibial plateau (asterisk). **e** A T2-weighted MR posterior coronal image shows a wide radial rupture of the medial meniscus (arrow), causing extrusion of the meniscus middle portion (arrowhead in **d**). **f** Sagittal T2-weighted image shows low signal intensity in a thick and extended subchondral bone marrow area adjacent to the condylar surface (arrow), corresponding to an area of degraded bone marrow. Under the tibial plateau, the thin horizontal band with a low signal intensity (arrowhead) is surrounded by an almost normal marrow signal intensity. These aspects are typical of a SIF for the tibial plateau and a SIF-ON for the condyle

To avoid confusion between entities that have a different clinical significance, in the rest of the manuscript, we will distinguish between “simple insufficiency fractures” (SIFs) and fractures complicated by irreversible collapse, referred to as “osteonecrosis after insufficiency fractures” (SIF-ONs) (Fig. 1). We will also use the term “Osteonecrosis of systemic origin” to designate epiphyseal osteonecrosis complicating ischemic lesions.

## Subchondral insufficiency fractures, without (SIF) or with osteonecrosis (SIF-ON)

The subchondral area of the epiphyses of the knees can be the site of SIFs, which are potentially transient, but can also become irreversible if evolving to osteonecrosis (i.e., SIF-ON) and collapse.

### Historical background

Spontaneous epiphyseal collapses (referred to as “idiopathic osteonecrosis of the knee”) have been described as a distinct entity by Ahlbäck et al. in 1968, with very precise descriptions of the clinical context [10]. With the advent of magnetic resonance imaging (MRI), it became evident that these collapses were preceded by areas of bone marrow edema (BME)-like signal changes, which were visible on MRI before radiographic collapse. Consequently, the term “spontaneous osteonecrosis of the knee (SONK)” has been extensively used to describe any spontaneous onset of pain that is associated with subchondral BME-like signal at MRI, or epiphyseal uptake on bone scans [11–13].

Other authors have noted similarities between spontaneous BME-like signal changes that are transient and those followed by focal subchondral osteonecrosis. These observations have led these authors to consider a common etiology and the hypothesis that an insufficiency fracture could represent the triggering event in both cases [14–22].

**Fig. 2:**
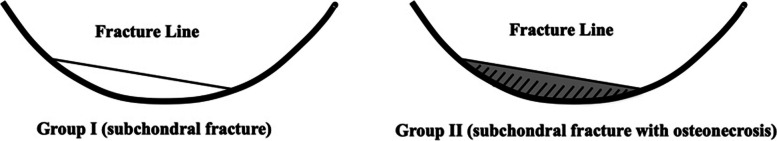
Diagram depicting the histopathologic classification of the two types of subchondral lesions, by Yamamoto and Bullough (with authorization of the author T.Y. [23]). In lesions previously considered as “spontaneous osteonecrosis of the knee (SONK),” the authors showed that the area located between fracture line and the articular contour contains either normal bone marrow (in subchondral insufficiency fractures (SIFs)), or focal osteonecrosis (in SIF complicated with osteonecrosis (SIF-ON))

A histological study by Yamamoto and Bullough in 2000 has largely contributed to advancing the debate. These authors retrospectively reviewed the histological material of surgically treated lesions that had been previously diagnosed as “SONK” based on the clinical presentation, imaging studies (including MRI), and pathologic findings. They found two types of lesions in histology: first, lesions that have a subchondral fracture line with no evidence of associated osteonecrosis, and second, lesions that have a subchondral fracture line associated with focal osteonecrosis (Fig. 2). Since the osteonecrotic area was strictly confined to the area between the fracture line and the articular surface, the authors concluded that the fracture is probably the primary event [23].

**Fig. 3:**
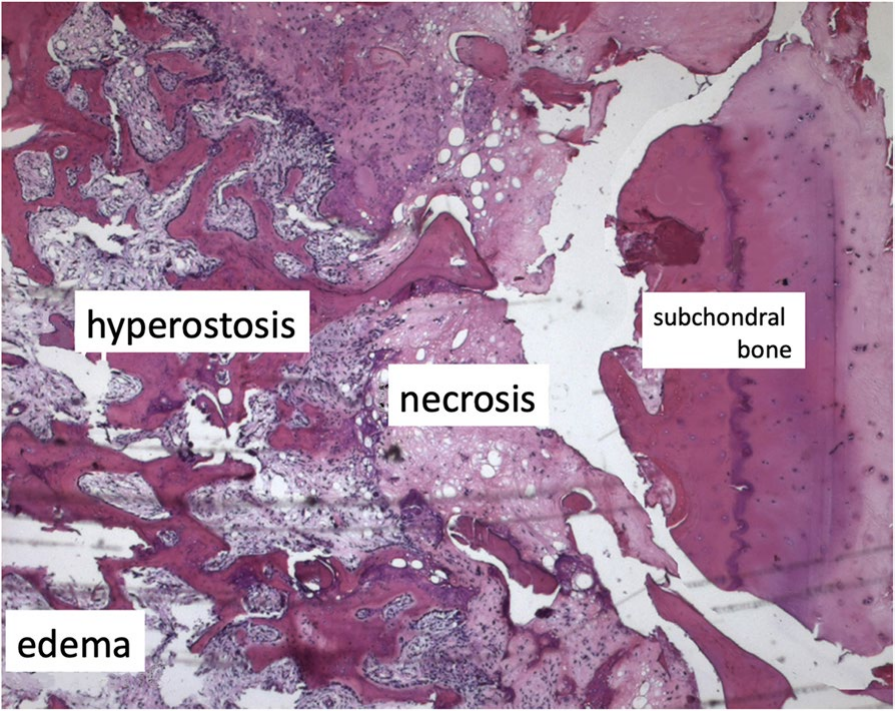
Histological pattern of a SIF. Bone marrow necrosis (“necrosis”) is located in the area between the fracture plane partially repaired (“hyperostosis”) and the subchondral bone (“subchondral bone”), detached in this case. Cellular marrow with edema ("edema") is located more proximally to the fracture plane

A few years later in 2008, another histological study of lesions diagnosed as “SONK” by Takeda et al. found that while in the early stages without collapse no osteonecrosis was seen, such features were visible in more advanced stages and confined to the area distal to the site of the fracture, which showed impaired healing [24] (Fig. 3).

### Clinical context

**Fig. 4:**
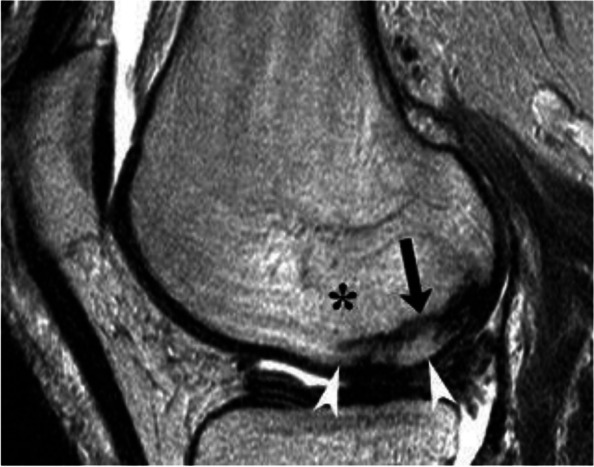
SIF in a patient with osteomalacia. T2-weighted MR image shows a low signal intensity band corresponding to a particularly thick trabecular fracture (arrow). The bone marrow of this portion of the condyle shows BME-like high signal intensity (asterisk). Note that the BME-like signal extends between the fracture and the bone-cartilage interface (arrowheads). That area of the subchondral bone is in continuity with the rest of the epiphyseal area

The clinical context of SIFs is the same as that of the so-called SONKs [25]. Both types of lesions occur mainly in patients above 60 years of age, mainly females, without any specific history of metabolic disorder or therapeutic intervention [6, 10, 25–28]. A potential link with underlying osteopenia is suggested by some studies, but not all [28–30]. However, it should be noted that insufficiency fractures may be particularly large or numerous in patients with conditions leading to bone fragility such as in renal transplant recipients or in cases of osteomalacia [18, 31] (Fig. 4).

The onset of symptoms is sudden in almost three-quarters of cases and occurs after a minor trauma or even after a wrong move. The onset is so sudden that patients commonly remember the exact moment it occurred [14, 26].

The lesions are predominantly located in the medial femoral condyle (in about 65% of cases), in the weight-bearing area [14, 26, 30]. The lateral condyle is much more rarely affected (in about 15% of cases), just as the medial tibial plateau. Involvement of the lateral plateau is very rare [28].

**Fig. 5:**
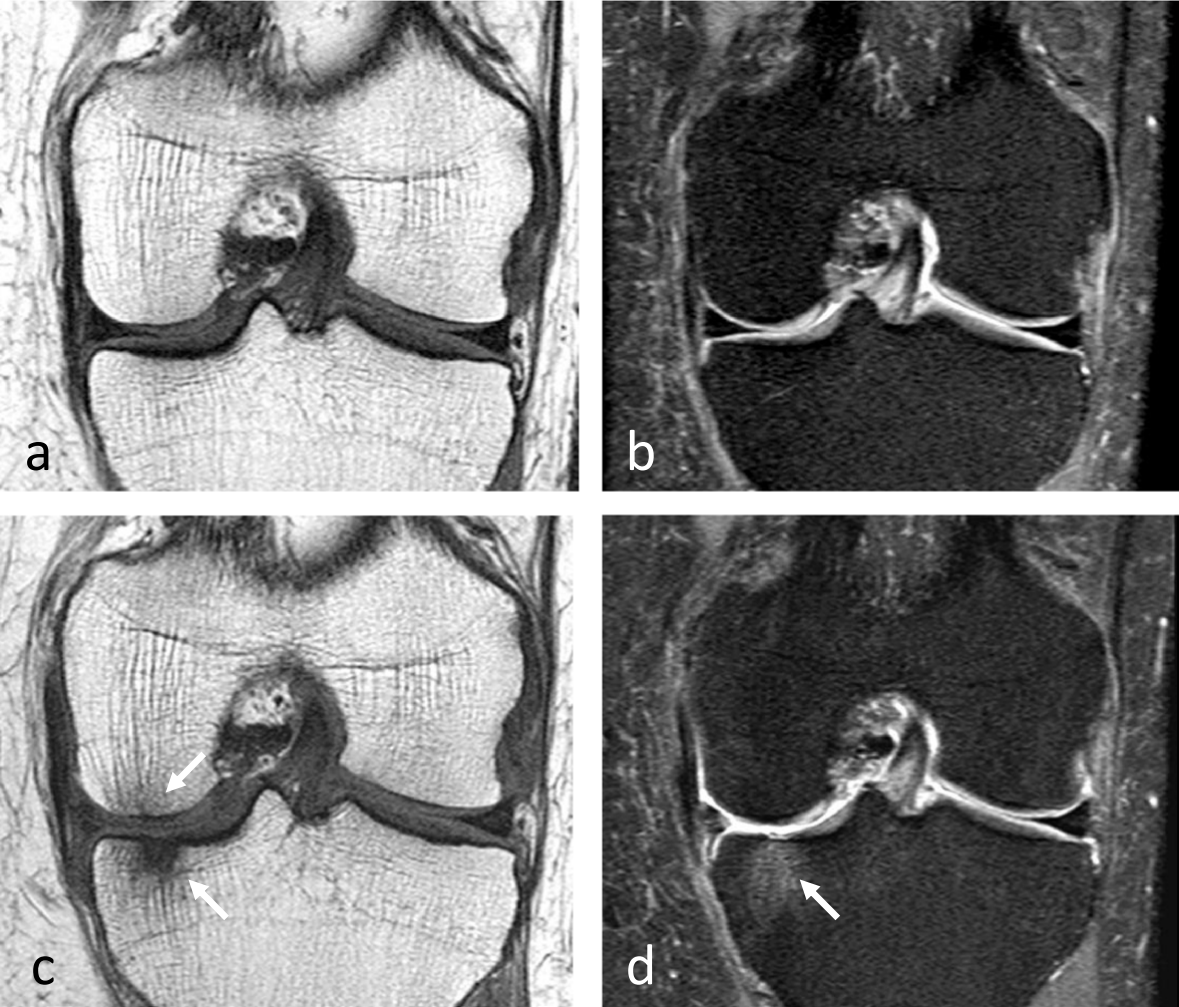
Consequence of a meniscectomy on a subchondral bone. **a**, **b** In this middle-aged subject, the coronal T1-weighted (**a**) and fat-suppressed T2-weighted (**b**) MR images show no abnormality. **c**, **d** Six months after resection of the (normal!) medial meniscus, T1-weighted (**c**) and fat-suppressed T2-weighted (**d**) MR images show a BME-like pattern in the lower pole of medial condyle and on the medial tibial plateau (arrows in **c** and **d**)

**Fig. 6:**
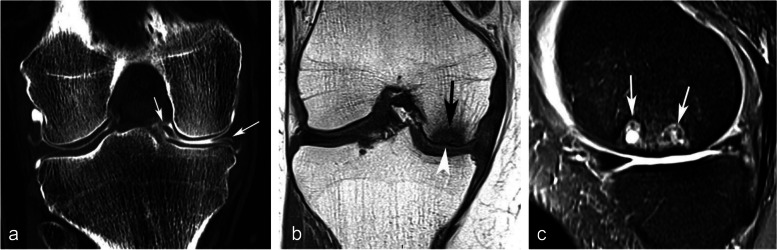
Bone decompensation after meniscectomy. **a** CT arthrography in a 72-year-old woman shows a horizontal tear of the medial meniscus body and a small fragment in the intercondylar notch (arrows). Ten months after meniscectomy, T1-weighted MR image (**b**) shows a very marked low signal intensity in the inferior pole of the medial condyle (arrow) and a small depression of the subchondral surface (arrowhead). Fat-suppressed T2-weighted image (**c**) shows extensive subchondral bone remodeling with heterogeneous high signal intensity in geode-like areas (arrows)

The association with chondrosis varies greatly depending on the series, from intact cartilage to severe chondrosis in cases with advanced collapse [7, 9, 30]. The association with a meniscal tear, on the other hand, has been consistently reported (in up to 76–94% of cases) [25]. In particular, the association with radial tears of the posterior horn of the medial meniscus or its attachment is found in more than 50% of cases [25, 27]. These meniscal tears are almost always associated with meniscal extrusions of 3 mm or more beyond the joint space margin [28] (Fig. 1d). They destabilize the meniscus and increase the mechanical strain on the articular surfaces by more than 25%, to the same extent as large meniscectomies [32] (Fig. 5). Similarly, subchondral fractures or osteonecrosis may also occur following meniscectomy [17, 25, 33–35] (Fig. 6).

### Radiographic appearance

**Fig. 7:**
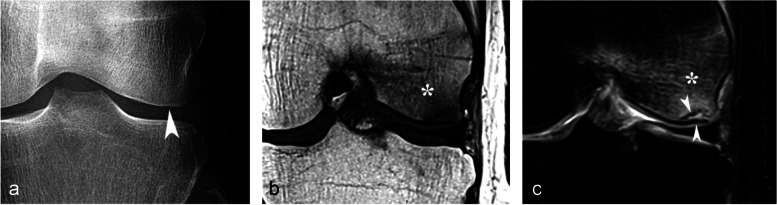
Early-stage subchondral insufficiency fracture (SIF). **a** Radiograph shows no significant finding apart from very subtle flattening of the condylar surface (arrowhead). **b** T1-weighted MR image shows moderate low signal intensity within the condyle (asterisk). **c** Fat-suppressed T2-weighted image shows BME-like high signal intensity more intense near the articular surface and extending to a part of the condyle without clear limits (asterisk). Note that the thin fracture line with low signal intensity is poorly visible with T1-weighting, and that with T2-weighting, high signal intensity extends within the area between the fracture and the articular surface (arrowheads). The high signal intensity in this area of the subchondral bone is an important finding to differentiate a SIF from a SIF-ON

**Fig. 8:**
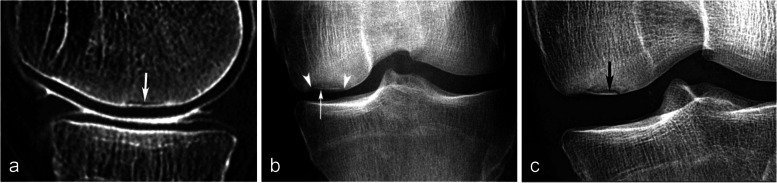
Radiological pattern of a “mobile” subchondral fracture, pathognomonic for necrosis. **a** On CT arthrography, a very thin fracture/separation in the subchondral trabecular bone appears as a radiolucent line (“eggshell dissection”) (arrow). **b** A few months later, a radiograph shows a collapse of the condyle lower pole (arrow) with breaks in the continuity of the subchondral bone plate (arrowheads). **c** An additional radiograph performed by fluoroscopy and with traction on the leg shows a slight opening of the subchondral bone dissection (arrow)

**Fig. 9:**
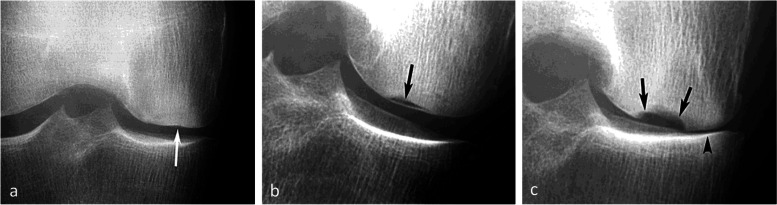
Evolution of an early subchondral insufficiency fracture with osteonecrosis. **a** Initial radiograph shows a slight hyperostosis near a discrete depression of the lower pole of the condyle (arrow). **b** Additional radiograph tangent to the posteroinferior pole of the condyle and performed with traction on the leg shows a typical subchondral bone dissection (arrow). **c** Three months later, this necrotic area shows a focal collapse (arrows), already complicated by a complete narrowing of the adjacent joint space (arrowhead)

For non-complicated insufficiency fractures (i.e., SIFs), radiographs are normal or show subtle abnormalities with subchondral radiolucencies and/or slight flattening of the convexity of the condyle (Fig. 7a) [23]. In the event of an insufficiency fracture associated with osteonecrosis (i.e., SIF-ON), the area of subchondral collapse becomes markedly heterogeneous and is surrounded by a sclerotic halo (Fig. 1b). At this stage, the articular surface may present signs of “macroscopic” fracture, with focal depression or disruption of the subchondral bone plate, or with separation of the subchondral bone plate adherent to the cartilage from the rest of the bone (showing as a subchondral radiolucent line—the crescent sign—or “eggshell” subchondral dissection) (Fig. 8). These signs are pathognomonic of osteonecrosis, whether related to an insufficiency fracture or systemic in origin. These abnormalities can be seen on radiographic views tangent to the area of interest, possibly performed in traction to open the subchondral separation (Fig. 9a and b). Later, secondary osteoarthritic changes become apparent (Fig. 9c) [23].

### MRI appearance

#### Classic appearance of BME-like signal

The key finding of SIFs is BME-like signal. However, this MRI sign is far from specific. At histology, actual BME (extracellular fluid) is rarely seen, and the MRI signal alterations originally described as edema are due to a variable amount of vascular dilatation, interstitial hemorrhage, cellular infiltration, granulation tissue, microfracture and callus formation, necrosis, and fibrosis, depending on the etiology [29, 36]. At imaging, it is therefore recommended to avoid the terms “edema” or “edematous signal” in favor of “edema-like signal.” In the rest of this article, we will refer to these MRI signal alterations of the bone marrow as “BME-like signal” or “BME-like pattern” [1, 9].

**Fig. 10:**
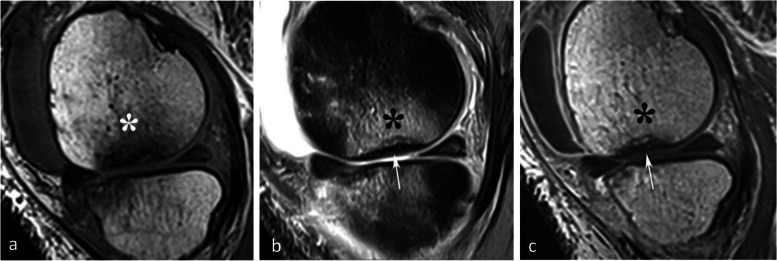
Enhancement pattern of a presumed subchondral insufficiency fracture with osteonecrosis. **a** T1-weighted MR image shows low signal intensity predominating near the condylar surface, extending deep in the epiphysis, and gradually attenuating with blurred outlines (asterisk). **b**, **c** On fat-suppressed T2-weighted image (**b**), this area (asterisk) shows high signal intensity and enhances after intravenous contrast injection (asterisk on post-contrast T1-weighted sequence in **c**). In this case, the subchondral area immediately adjacent to the bone plate (arrows) shows low signal intensity with fat-suppressed T2-weighting (in **b**) and no enhancement with post-contrast T1-weighting (in **c**). This pattern corresponds to an area of degraded subchondral fatty marrow, suggestive of a SIF-ON

BME-like signal refers to an ill-defined area of bone marrow with moderately decreased signal intensity on T1-weighted images and with increased signal intensity on T2-weighted images, more evident on fat-suppressed fluid sensitive images (Fig. 7b, c) (i.e., fat-suppressed T2 (T2FS), short-tau inversion recovery (STIR), fat-suppressed proton density (DPFS), Dixon T2 “Water” images). For the sake of simplicity, we will refer to all types of fat-suppressed fluid-sensitive sequences as “T2FS.” After intravenous injection of contrast media, the signal on T1-weighted sequences enhances and becomes practically similar to that of normal bone marrow (Fig. 10) [8, 22, 37].

The location and extension of BME-like signal changes vary depending on their origin. In SIFs, the signal abnormality predominates near the articular surface and extends in a gradient over a variable portion of the epiphysis. On the other hand, BME-like changes in osteoarthritis are generally more confined [25]. The extension of the BME-like signal has no prognostic value [25].

**Fig. 11:**
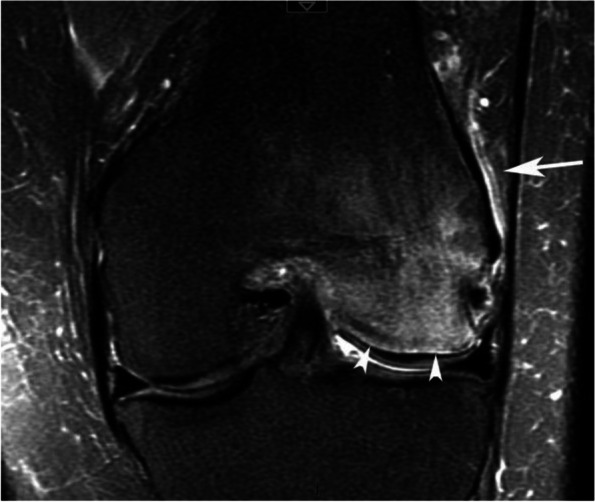
Other features associated with bone-marrow-like signal. In case of intense BME-like signal intensity, the area immediately adjacent to the subchondral bone plate may show a very intense, finely linear high signal on fat-suppressed T2-weighted images (arrowheads), which could be linked to hyperemia of this richly vascularized area. Edema-like high signal intensity may also involve the neighboring soft tissues (arrow)

BME-like pattern may be accompanied by a thin high signal intensity line on T2FS images, immediately adjacent to the subchondral bone plate, which could be related to hyperemia in this richly vascularized area [38]. In cases of SIF, this aspect is present in more than half of cases, especially in the acute (< 3 months) or subacute (3–6 months) phase [39]. In addition to the intraosseous BME-like signal, adjacent soft tissue edema signal may also be seen and has been reported in the vast majority of SIFs involving a femoral condyle (Fig. 11) [28].

Importantly, the BME-like signal in SIF has poorly defined borders, opposite to osteonecrosis of systemic origin where the lesion is surrounded by a distinct, geographic rim of sclerosis/low signal intensity line [20, 21, 27, 40].

#### Visibility of the fracture line and its hypothetical pathogenic implication

**Fig. 12:**
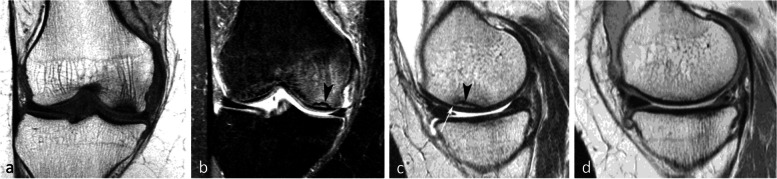
Visibility of subchondral insufficiency fracture (SIF) in a BME-like area. **a**–**c** On T1-weighted MR images (**a**), the fracture can be masked by the neighboring low signal intensity, while on fat-suppressed T2-weighted (**b**) or T2-weighted images (**c**) the fracture is apparent (arrowheads), contrasting with the surrounding high signal. In this case of SIF, BME-like high signal intensity is present on both sides of the fracture, including the area between the latter and the articular surface, corresponding to the area with infiltrated but a not degraded fatty marrow. Note that the fracture line does not completely isolate this subchondral area. The potential for this SIF to heal was confirmed by a follow-up study a few months later showing normalization (**d**)

When visible, the fracture has the appearance of a thin, somehow curvilinear line located at a short distance from the articular surface. This line shows low signal intensity on all sequences and is best visible in T2FS images because on T1-weighted images, it can be masked by the adjacent low-intensity BME-like signal changes. Importantly, in SIFs, the BME-like pattern is present on both sides of the fracture line (Figs. 7c and 12).

**Fig. 13:**
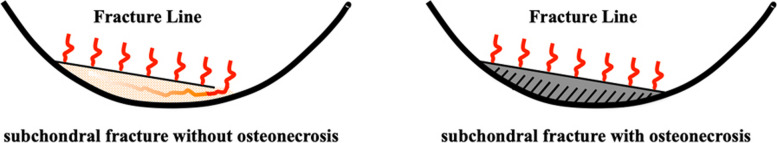
Modified diagram from Yamamoto and Bullough. Based on the authors’ concept, this modified diagram (adapted from Fig. 2) proposes that a SIF (subchondral insufficiency fracture) has the potential to heal provided that there is continuity between the area located between the fracture plane and the articular surface and the rest of the medullary epiphyseal space. Conversely, a SIF-ON would occur in case this continuity is lost

One possible pathogenic hypothesis explaining the difference between a SIF that evolves towards a SIF-ON and a SIF that heals may be related to how the fracture line isolates completely or not the adjacent subchondral area (Fig. 13). The presence of a complete fracture line isolating entirely the subchondral bone between the fracture and the articular surface may compromise the vascularization of the isolated subchondral area, possibly leading to necrosis. On the other hand, if the fracture line does not isolate completely an area of subchondral bone, a continuity persists with the rest of the epiphysis, and the vascularization may be preserved in this area, preventing osteonecrosis (Fig. 12c and d).

**Fig. 14:**
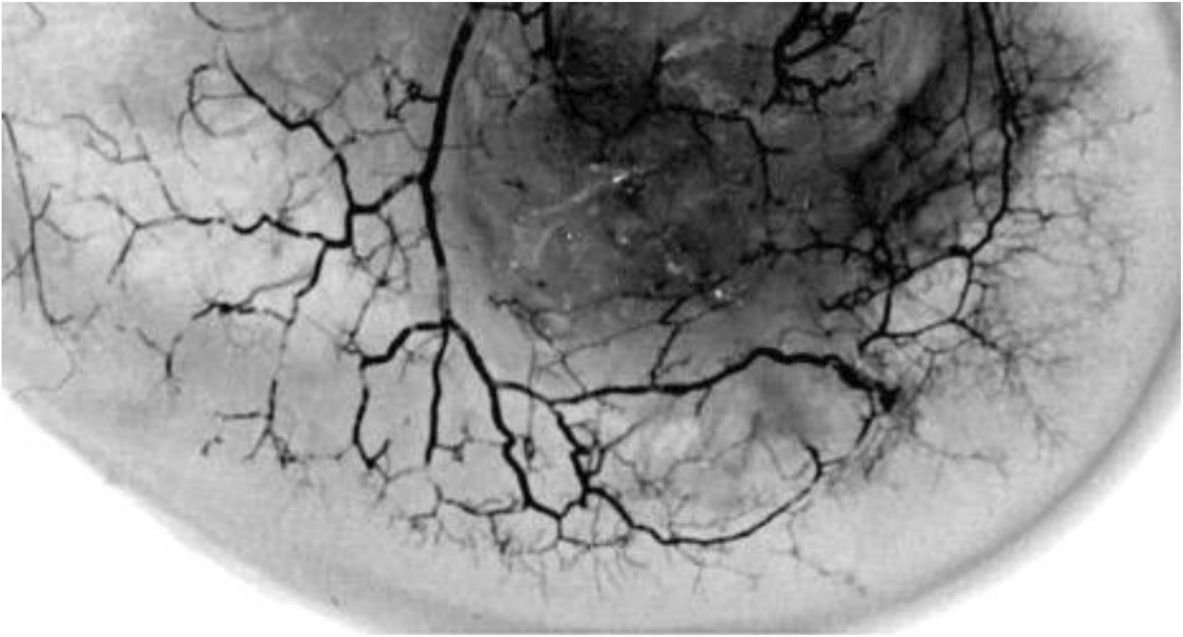
Microvascularization of the subchondral bone of a condyle (from Reddy and Frederick [41]). The subchondral area has multiple anastomoses between terminal arterioles

This pathogenic hypothesis is based on the assumption that the normal vascular supply of the epiphyseal subchondral bone results from a network of anastomotic terminal arterioles. This seems to be well-illustrated in an anatomical study by Reddy and Frederick (Fig. 14) [41]. But, even in case of persistent communication between the remaining epiphysis and the subchondral bone adjacent to the fracture, there may still be inadequate blood flow in certain cases, especially if the area is too large to be fully supported by the remaining micro-arterial network.

Another hypothesis could be that necrosis occurs following pseudarthrosis of the insufficiency fracture, resulting in the separation of a distal bone fragment that has become unstable, with the loss of its blood supply ultimately leading to osteonecrosis [24]. But this hypothesis does not account for the subchondral bone signal alterations (low signal on T2-weighted and post-contrast T1-weighted images) which can be observed in some early lesions without collapse or subchondral separation (see below).

#### Evolution of SIFs

In SIFs, conservative treatment with protected weight-bearing generally allows a reduction in pain and healing without sequelae [3, 4, 26, 42]. The treatment generally consists of protected weight-bearing for a period of 6 weeks using crutches, followed by gradual weight-bearing using walking sticks. Physiotherapy can help prevent muscle atrophy. Adjuvant treatment with bisphosphonates is suggested by some [43, 44]. In uncomplicated SIFs, the BME-like signal normalizes within a few months (Fig. 12).

#### Evolution of SIF-ONs

**Fig. 15:**
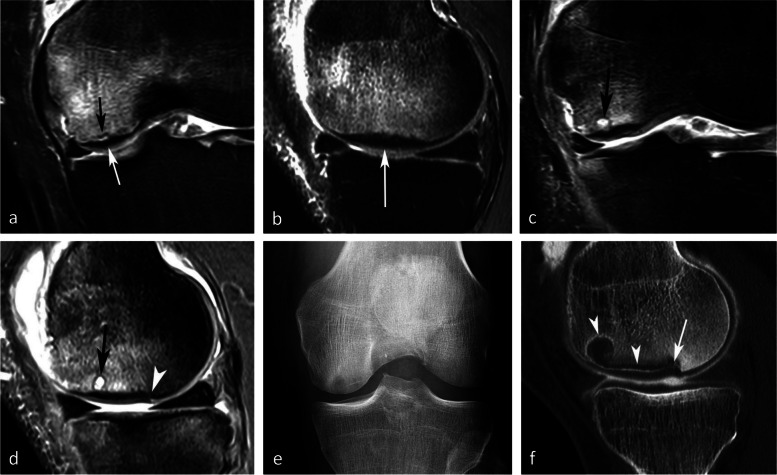
Example of a pejorative evolution of a SIF to a SIF-ON. **a**, **b** At the initial stage, the BME-like high intensity on fat-suppressed T2-weighted MR image and a small fissure line (black arrow in **a**) are suggestive of a SIF. However, the subchondral bone adjacent to the cartilage shows a thick and extensive low signal-intensity area (white arrows in **a** and **b**). **c**, **d** Five months later (**c**, **d**) the marrow abnormalities persist, with the appearance of small geode-like images (arrows) and a small break in the subchondral bone plate (arrowhead in **d**). **e**, **f** Three months later, radiograph (**e**) still shows heterogeneous density of the lower condylar pole, and CT arthrography (**f**) shows a subchondral geode and bone resorption (arrowheads), associated with a focal collapse of the subchondral bone surface (arrow)

A marked collapse of the articular surface indicates decompensation of the lesion. The subchondral bone under the collapsed articular surface may become disorganized and present various aspects (Fig. 15). Later in the disease, subchondral fractures can turn into transchondral fractures releasing fragments from the articular surface.

**Fig. 16:**
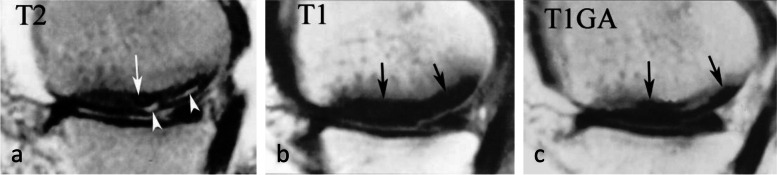
Subchondral insufficiency fracture complicated with osteonecrosis (SIF-ON) and early subchondral collapse. **a** T2-weighted MR image shows a wide subchondral low signal intensity area (arrow). On T1-weighted images without (**b**) and after contrast injection (**c**) a large portion of this area does not enhance after intravenous contrast injection (arrows in **c**). Note also a thin line with high fluid-like signal intensity in dissected subchondral fracture on T2-weighted image (arrowhead in **a**)

Even before the macroscopic fracture stage, the bone marrow immediately adjacent to the subchondral bone may show decreased signal on T2w images [13, 20, 21, 40, 45–48]. This low signal intensity bone marrow may correspond histologically to degraded, “saponified,” solidified marrow fat [49]. This area shows no enhancement on post-contrast T1-weighted images (Fig. 16) [20, 40, 47]. When it is thin, this area of low intensity on T2-weighted images can be difficult to distinguish from a thickened subchondral bone plate [48].

#### Prognostic value of bone marrow low signal intensity on T2-weighted or post-contrast T1-weighted images

**Fig. 17:**
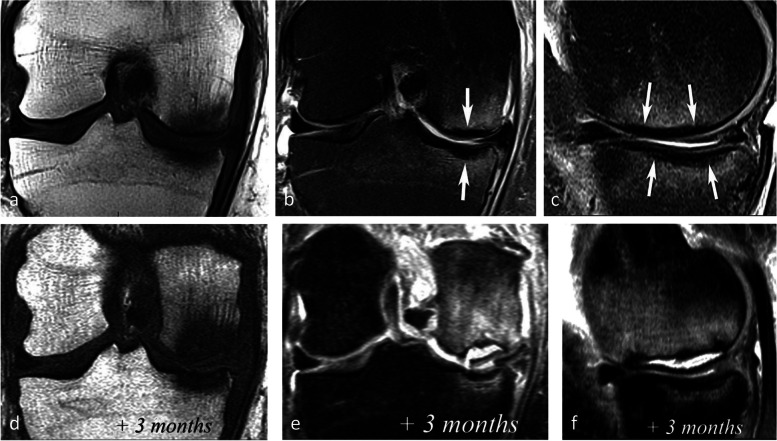
Negative prognostic value of low signal bone marrow on T2-weighted MR image. **a** The subchondral bone of the two medial femorotibial joint surfaces shows marked low signal intensity on a T1-weighted MR image. **b**, **c** Coronal and sagittal fat-suppressed T2-weighted MR images (**b**, **c**) show relatively wide and thick subchondral areas adjoining the articular surface, with low signal intensity (arrows), suggestive of SIF-ON. **d**–**f** Three months later, a significant bone marrow low signal intensity persists on T1-weighted image (**d**) and coronal and sagittal fat-suppressed T2-weighted images show pejorative evolution with a collapse of the tibial plateau and a wide subchondral bone dissection and chondral fracture in the lower pole of the condyle (**e**, **f**)

**Fig. 18:**
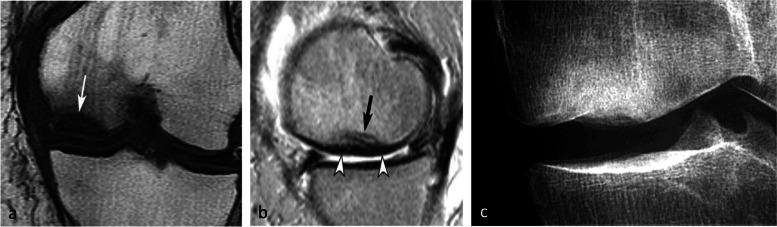
Early subchondral insufficiency fracture with osteonecrosis (SIF-ON) and collapse. **a** T1-weighted MR image shows an area of low signal intensity in the inferior pole of the medial condyle (arrow). **b** T2-weighted image (**b**) shows an immediate subchondral low signal intensity area longer than 14 mm (arrowheads), suggestive of early SIF-ON. Note also several fissure lines away from the surface (arrow), which is also a risk factor for an evolution towards a SIF-ON. **c** A few years later, a radiograph shows an irregular collapsed articular surface, confirming the initial suspicion of SIF-ON

The pejorative prognostic value of bone marrow low signal on T2-weighted images was described as early as in 1990 [13]. Subchondral areas of low T2 signal intensity thicker than 4 mm or longer than 14 mm were shown to be predictive of irreversible lesions, with sensitivities/specificities of about 100%/80% for the thickness and nearly 90%/90%, for the length, respectively (Fig. 17) [20, 21]. Subchondral bone marrow areas of more than 3 cm^2^ not enhancing on post-contrast T1-weighted images were also shown to be a factor of poor prognosis [40]. Other poor prognostic factors include a clear deformation of the articular surface and the location of fracture lines far from the articular surface (Fig. 18) [21].

On the other hand, a subchondral low T2 signal intensity area thinner than 2 mm is usually indicative of a reversible lesion [20, 21]. In this case, this low signal intensity area most likely corresponds to a very limited area of superficial osteonecrosis, or to granulation and fracture repair tissue.

**Fig. 19:**
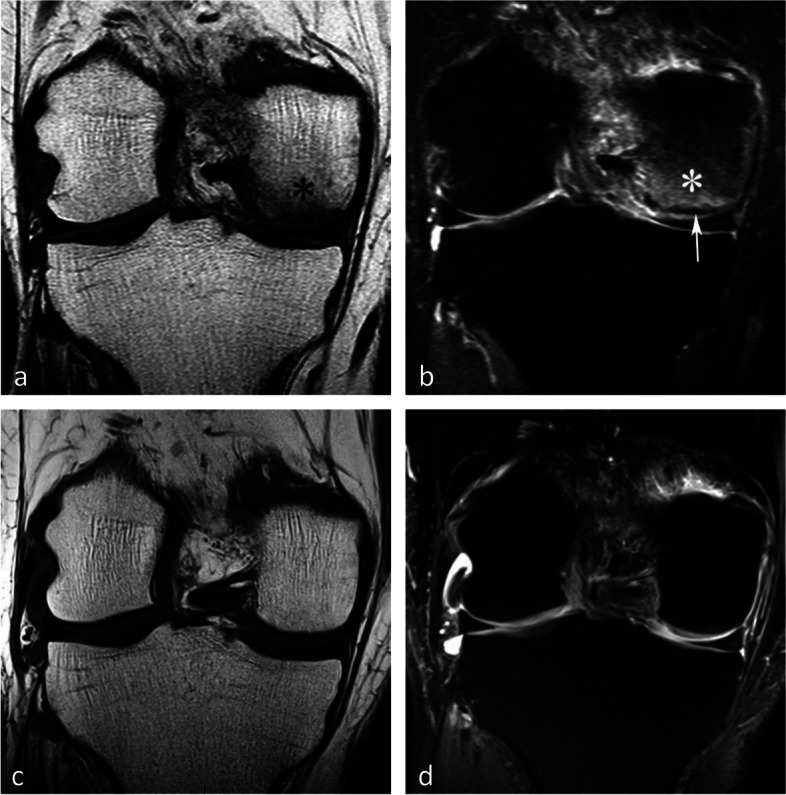
Borderline thickness of bone marrow low signal on T2-weighted image. **a**, **b** MRI shows low signal intensity on T1-weighted (**a**) and high signal intensity on fat-suppressed T2-weighted images in the medial femoral condyle (**b**) (asterisks). Immediately near the subchondral surface, fat-suppressed T2-weighted image shows a very thin layer of tissue of borderline thickness with low signal intensity (arrow in **b**). **c**, **d** In the present case, the follow-up at 3 months showed healing, with normalization of the signal in the inferior pole of the condyle

Lesions with subchondral low T2 signal intensity areas between 2 and 4 mm should be considered of uncertain prognosis and be followed up (Fig. 19).

**Fig. 20:**
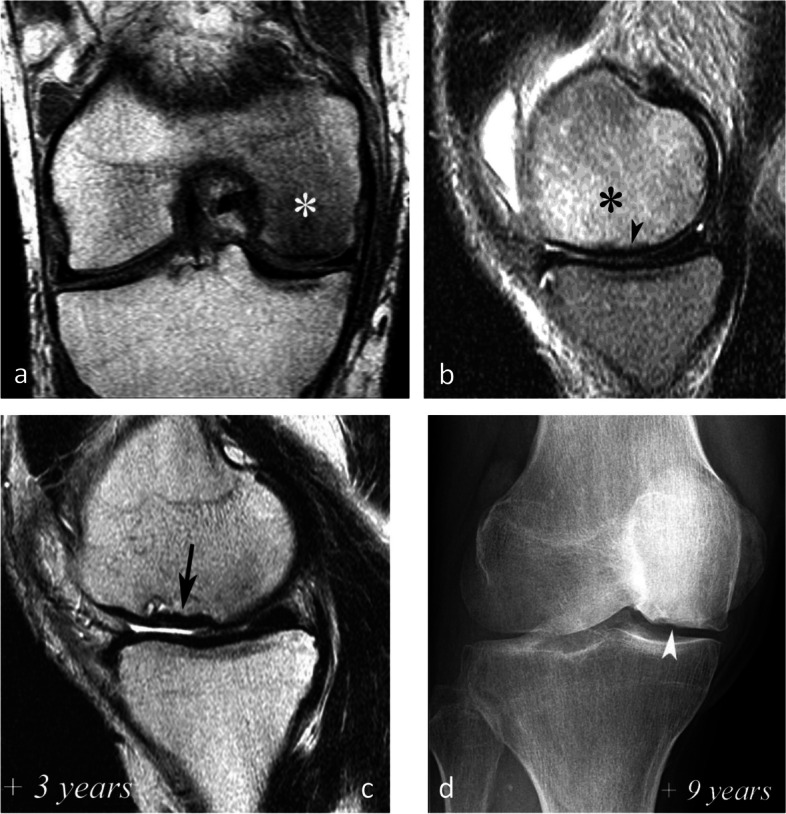
Evolution in two stages of subchondral insufficiency fracture (SIF). **a**, **b** Coronal T1-weighted (**a**) and sagittal T2-weighted (**b**) MR images show BME-like signal intensities in medial condyle (asterisks), except for a very thin (< 4 mm in thickness) subchondral low signal intensity area on the T2-weighted image (arrowhead in **b**). **c** MRI follow-up three years later with T2-weighted image (**c**) shows a small deformation of the lower pole of the condyle with thickening of the subchondral low signal intensity area, now exceeding 4 mm in thickness (arrows), suggesting the evolution of the SIF towards osteonecrosis (SIF-ON). **d** Nine years after the initial examination, a radiograph confirms an irreversible lesion, with deformation of the epiphyseal surface (arrowhead), which is moderate in this case and clinically well tolerated

However, these prognostic factors are merely indicative, and an initially uncomplicated SIF may still decompensate at a later stage (Fig. 20), either spontaneously or following a destabilizing meniscectomy.

**Fig. 21:**
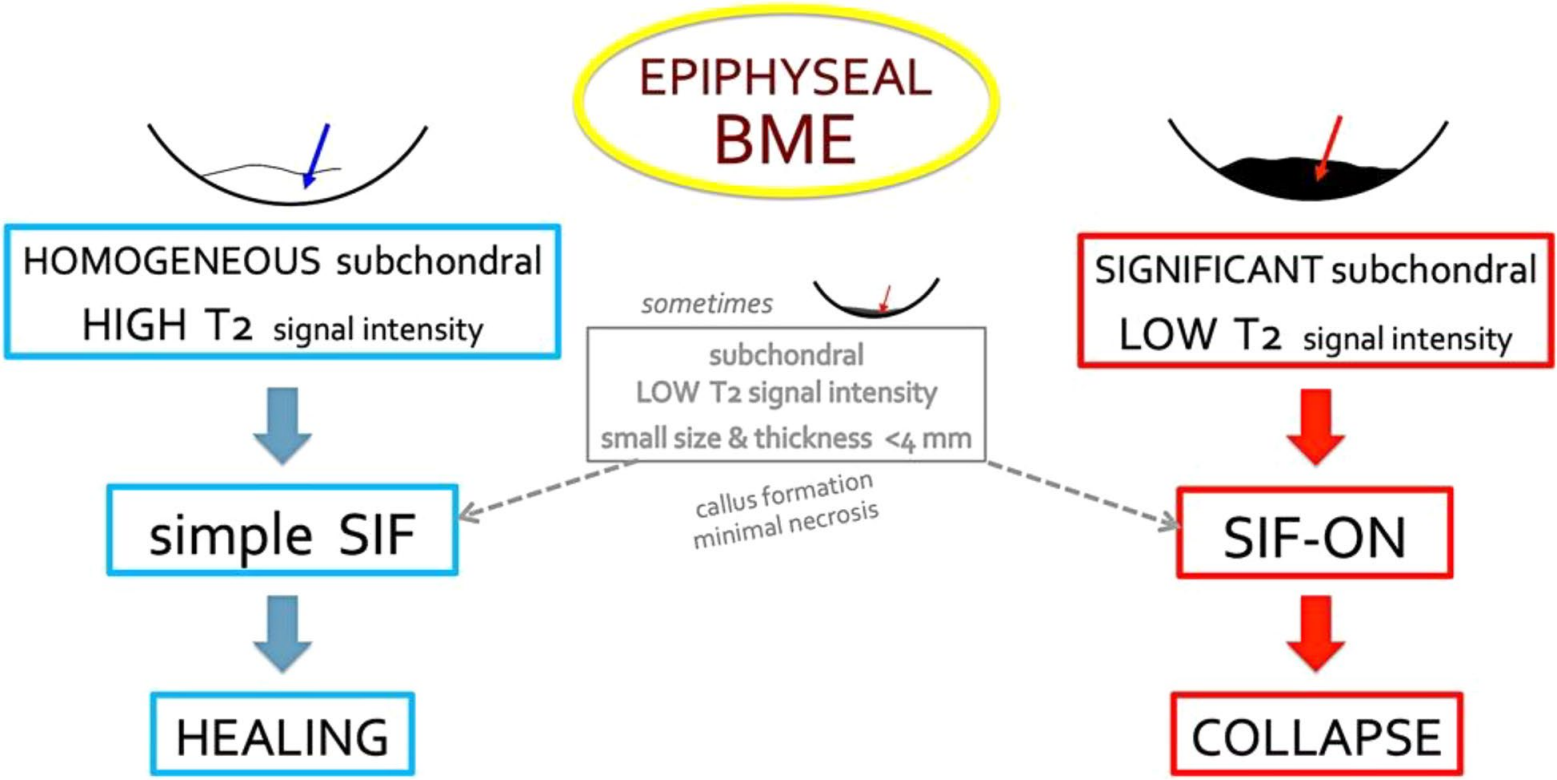
Diagram summarizing the prognostic value of typical subchondral signal intensity on T2-weighted sequences in SIF and SIF-ON. Note that in some cases, a very limited area with subchondral low T2 signal intensity is of doubtful prognosis and may progress either to healing or to SIF-ON. Without a particular event (meniscectomy or rapid cartilage degeneration for example), the evolution of a typical SIF towards a SIF-ON is very rare

The prognostic value of T2-weighted MR images is summarized in Fig. 21.

### Late evolution of lesions

The occurrence of complications depends on multiple factors, including the patient’s weight, degree of osteopenia, treatment delay, the extent of chondrosis, and degree of meniscal extrusion associated with a radial tear or root tear of the posterior horn of the medial meniscus [4, 7, 28].

**Fig. 22:**
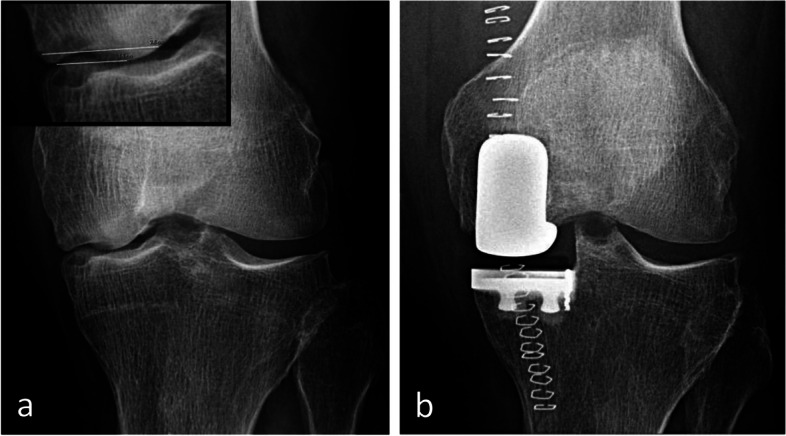
Evolution of subchondral osteonecrosis related to insufficiency fracture (SIF-ON) according to the size of the necrosis. **a** Radiograph of this SIF-ON shows a collapse of the inferior pole of a condyle, which extends to more than 50% of its width (64%). Its pejorative evolution led to the placement of prosthesis a few months later (**b**)

**Fig. 23:**
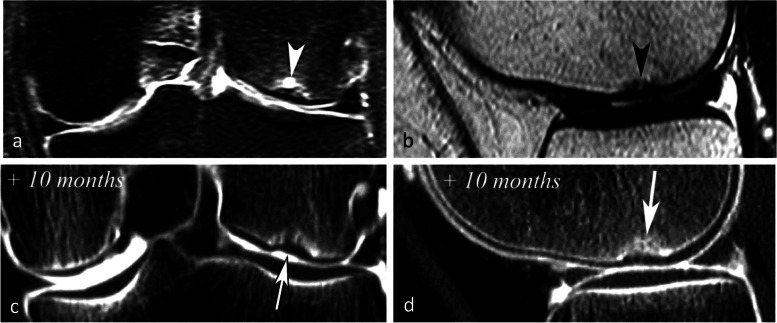
Evolution of subchondral osteonecrosis related to insufficiency fracture (SIF-ON) according to the size of the necrosis. **a**, **b** Fat-suppressed T2-weighted MR coronal images (**a**) and T2-weighted sagittal image (**b**) show a very small heterogeneous signal intensity area (arrow). **c**, **d** Ten months later, CT arthrography images show very limited collapse (arrows)

The evolution towards a progression of the articular surface collapse is obviously related to the size of the necrotic area. For example, the prognosis is unfavorable when the width of the area of necrosis is greater than 40–50% of the width of the condyle (Fig. 22) or when its area is greater than 5 cm^2^, while smaller collapses may stabilize spontaneously [16, 26, 30] (Fig. 23). The prognostic value of the dimensions of the lesion is also important for the tibial plateaus [50].

## Other causes of bone marrow edema-like signal intensity

Many other conditions can cause a BME-like signal in a knee, for example in reaction to inflammatory disorders (osteitis, arthritis), inflammatory benign tumors (e.g., osteoid osteoma, chondroblastoma, etc.), or malignant tumors, with clinically distinct presentations. Sometimes the cause is obvious, such as post-traumatic bone contusions, which disappear after a few weeks or months [51]. In other cases, the cause is a transient or non-transient reaction to a chronic condition (osteoarthritis for example) or part of a condition of unknown origin referred to as the complex regional pain syndrome type 1.

### Complex regional pain syndrome type 1 (CRPS 1)

Formerly called algodystrophy or reflex sympathetic dystrophy, CRPS 1 is a polymorphic disorder, without a specific biological, bacteriological, or anatomical substrate, of which the presumptive diagnosis is based on a combination of clinical and imaging criteria including local or loco-regional pain, cutaneous hyperesthesia, vascular disturbances, local or loco-regional edema, radiological bone rarefaction, and increased or decreased bone activity at bone scan [52].

The condition can be triggered by anything (including trauma, surgery, insect bites…), or it can occur without any identifiable trigger at all [52]. Its etiology remains unknown. It has been suggested that a local ischemic episode may initiate a chain of events resulting in BME, but this hypothesis lacks convincing arguments [53]. The pathogenesis of pain is poorly understood and probably multifactorial: neurovegetative dysregulation, increased intraosseous pressure with irritation of sensory nerves within the bone, and increased bone turnover with or without microfractures [53].

The clinical criteria have poor specificity and there is no “reference standard” to formally establish the diagnosis. Pain is usually out of proportion at physical examination. But pain and edema are not sufficient for the diagnosis, and the presence of other sensory, vasomotor, sudomotor, trophic, and motor criteria is required to improve specificity (“Budapest criteria”) [54]. Ultimately, this remains a diagnosis of exclusion, which is why imaging can be a valuable tool in confirming or ruling out other potential causes [53].

In the radiological literature, this entity is also referred to as “transient osteoporosis,” because osteopenia is usually seen on radiographs, or “migratory osteoporosis” when the condition moves between joints. At MRI, BME-like signal changes are seen, preceding the radiographic changes; therefore, the term “transient bone marrow edema syndrome” has been used [1, 9].

**Fig. 24:**
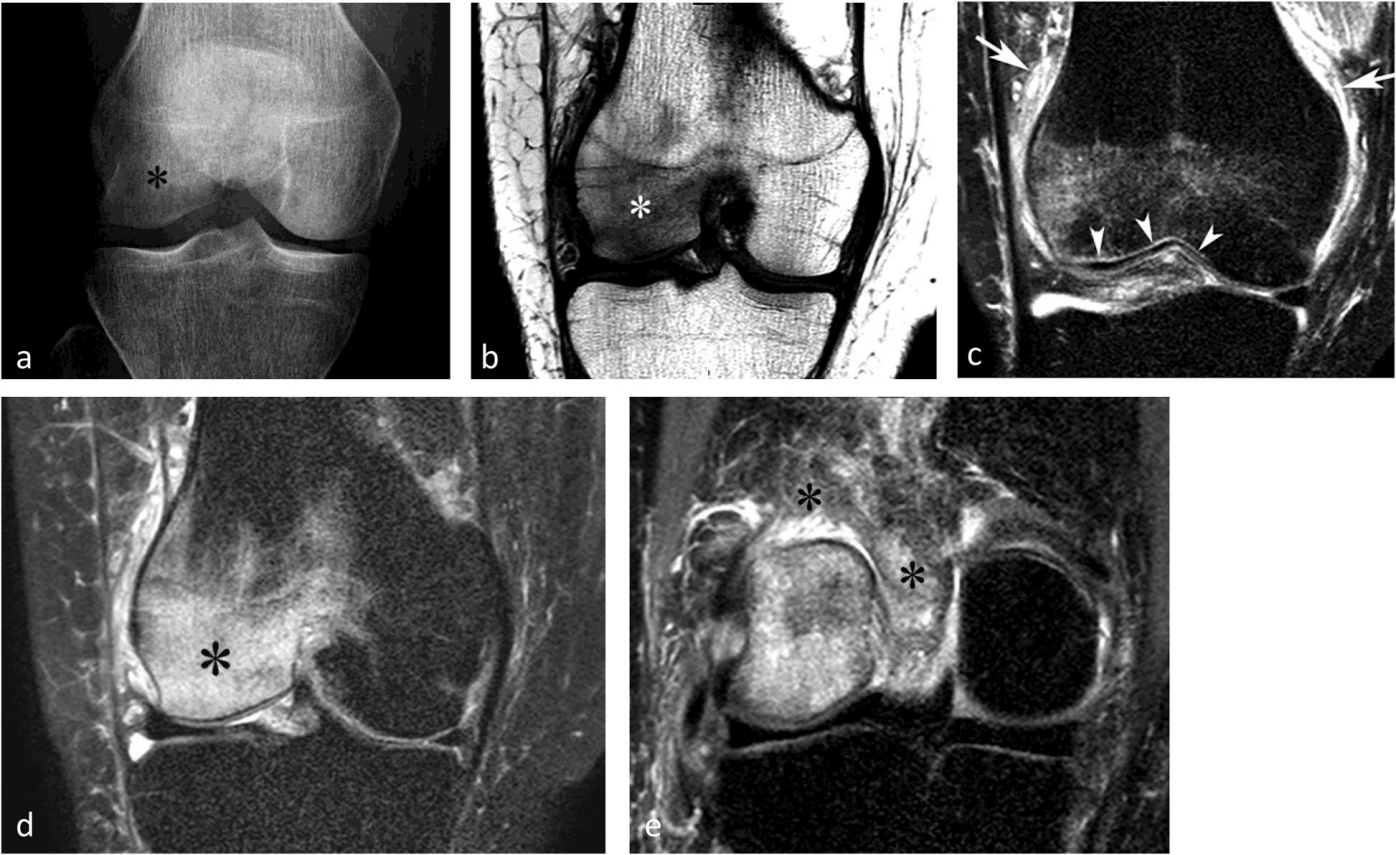
Typical pattern of complex regional pain syndrome type I (CRPS1). **a** Radiograph shows intense bone loss in the lower pole of a condyle (asterisk). **b**–**e** T1-weighted (**b**) and fat-suppressed T2-weighted (**c**–**e**) MR images show a BME-like pattern similar to that of a SIF (asterisks in **b** and **c**). Note the particularly intense edema-like signal intensity also in the soft tissues (arrows in **c** and asterisk in **e**), as well as a very fine linear area with intense high signal intensity, immediately adjacent to the subchondral bone plate (arrowheads in **c**)

The typical radiographic appearance is increased bone transparency, sometimes heterogeneous (“speckled” or “dappled”), visible from 1 to 3 months after the onset of symptoms and which can, in the knee, persist for months. This increased bone transparency may only affect a portion of the anatomical area, particularly in early lesions (Fig. 24a) [55].

On MRI, the alterations are similar to those seen in most transient epiphyseal lesions: BME-like signal changes which are more or less extensive, predominate near the articular surfaces, and are associated with edema-like signal in the adjacent soft tissues (Fig. 24c-e) [56]. A fine high-intensity line is also frequently present immediately adjacent to the subchondral bone lamina on T2FS images (Fig. 24c) [39].

**Fig. 25:**
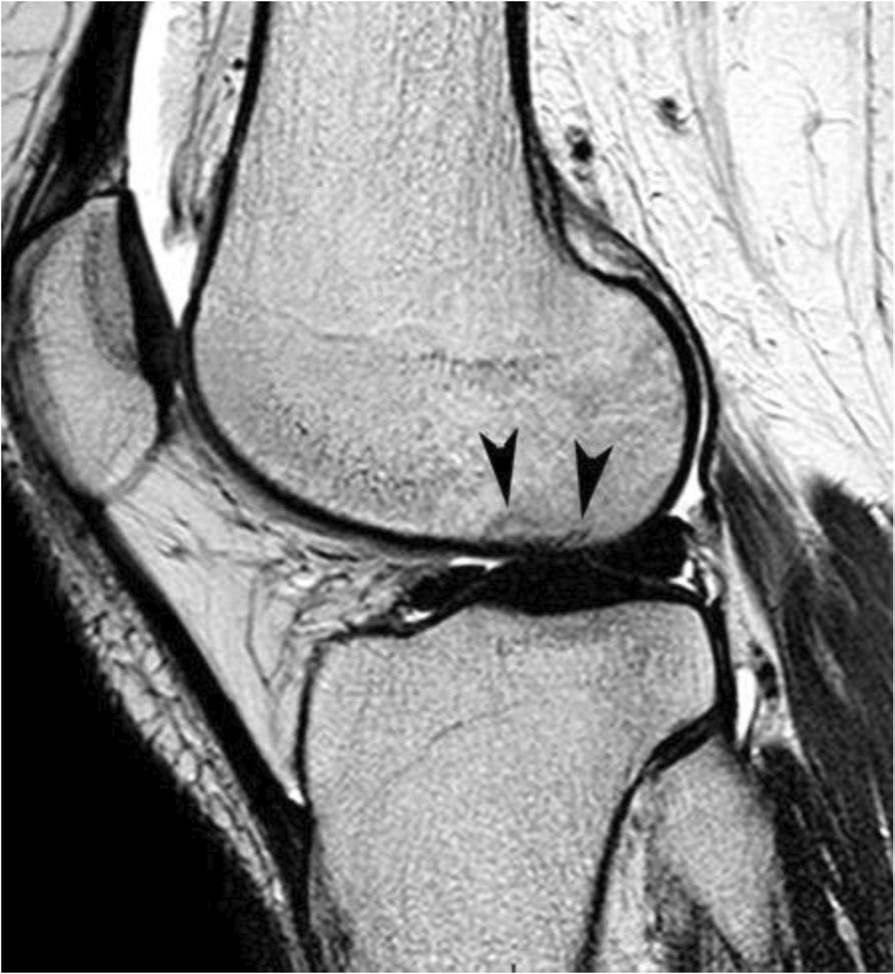
Trabecular fractures in case of CRPS1 (same MR examination as the previous figure). T2-weighted sagittal MR image shows thin, incomplete low-intensity linear images (arrows), as in a subchondral insufficiency fracture

MRI changes in the bone marrow precede the radiographic bone rarefaction by several weeks and may regress before it. Therefore, in the case of chronic CRPS 1, the MRI may be normal, contrasting with radiological and clinical alterations [53, 57].

In practice, there is no notable difference between the MRI appearance of CRPS 1 and that of other transient epiphyseal lesions, except that in CRPS 1 edema-like alterations in the bone and soft tissues are often more intense and last longer than in SIF. To add to the confusion, subtle deformations of the subchondral surface and thin subchondral fracture lines may also be seen in CRPS 1 (Fig. 25) [58, 59].

**Fig. 26:**
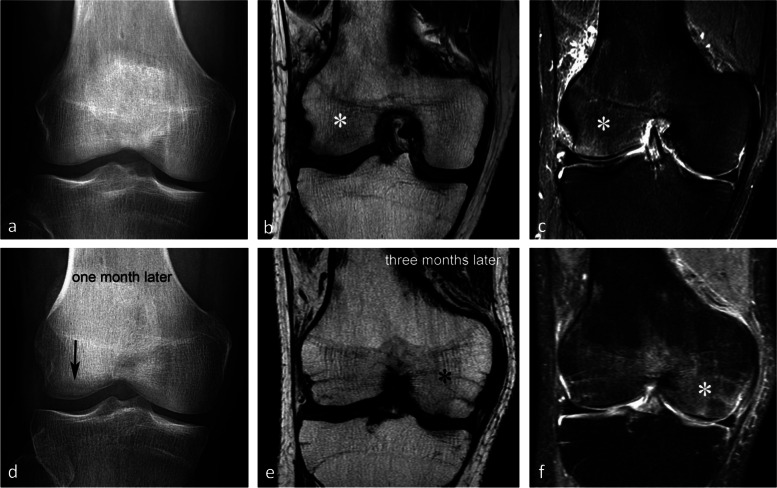
Migrating bone marrow edema (BME)-like lesion in CRPS1. **a** In early phase, the radiograph (**a**) was normal. **b**, **c** However, MR images showed typical BME-like signal appearing as low and high signal intensity on T1- (**b**) and fat-suppressed T2-weighted (**c**) images, respectively, in the lateral condyle (asterisks). **d** One month after the initial examination, radiograph show bone loss at the same location (arrow). **e**, **f** Three months after the initial examination, T1- and fat-suppressed T2-weighted images (**e** and **f**) show almost normal lateral condyle, while BME-like signal intensities have appeared in the medial condyle (asterisks)

The migration of the BME-like pattern from one portion of the epiphysis to another or from one epiphysis to another can be observed at follow-up imaging. This migration of the BME-like pattern is the only definitive characteristic at MRI that distinguishes CRPS 1 from SIF (Fig. 26) [56, 60, 61].

**Fig. 27:**
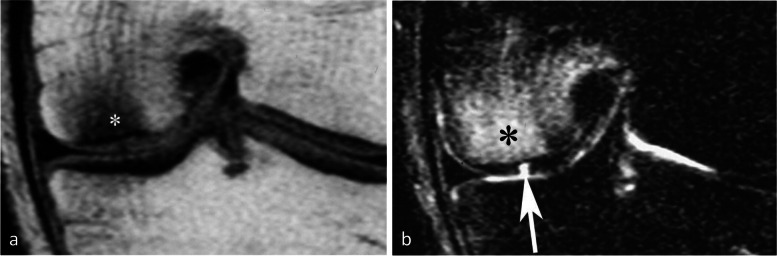
Bone marrow edema (BME)-like signal associated with focal chondral lesion. **a**, **b** T1-weighted (**a**) and fat-suppressed T2-weighted MR images (**b**) show BME-like signal intensities similar to those of subchondral insufficiency fractures (SIFs) (asterisks). Here, the BME-like signal intensity is related to a deep focal cartilage defect (arrow in **b**), evident in the present case

In summary, the MRI appearance of CRPS 1 lesions is almost similar to that of SIF, apart from the usually greater intensity of BME-like signal changes and the frequent migration of lesions at follow-up.

### BME-like signal associated with chondral lesions

The so-called BME-like lesions are frequently seen in osteoarthritis and are typically associated with pain [62]. At histology, these lesions correspond to a number of abnormalities, including bone marrow necrosis, bone marrow fibrosis, and necrotic or remodeled trabeculae, but edema is not a major constituent [63]).

**Fig. 28:**
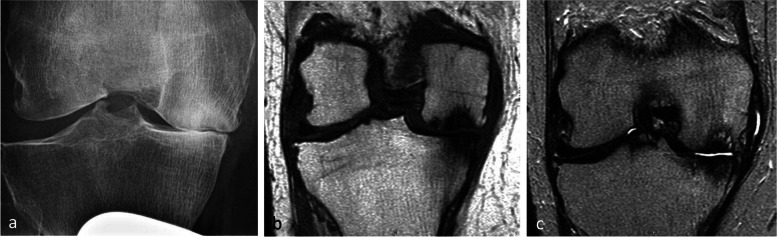
Subchondral bone changes associated with advanced osteoarthritis. **a** Radiograph shows heterogeneous subchondral bone densification and complete narrowing of the medial joint space. **b**, **c** T1- and T2-weighted MR images (**b** and **c**) show focal heterogeneous low signal intensities of these areas, quite similar to what could be seen in subchondral osteonecrosis

BME-like signal changes are generally less intense and less extensive in osteoarthritis than in SIF and their location is more variable [64]. BME-like signal can either be homogeneous (Fig. 27) or heterogeneous, especially in cases of advanced osteoarthritis, due to associated structural bone changes (hyperostosis, necrosis, geodes, etc.) (Fig. 28) [8].

It is therefore possible to confuse BME-like lesions associated with osteoarthritis, with that associated with SIF or SIF-ON, particularly since the latter is also found in the elderly population, who often present with a certain degree of chondrosis. This confusion is evident in some studies on lesions described as SIFs, where knees with complete cartilage destruction and those with intact cartilage are included in the same series [7].

**Fig. 29:**
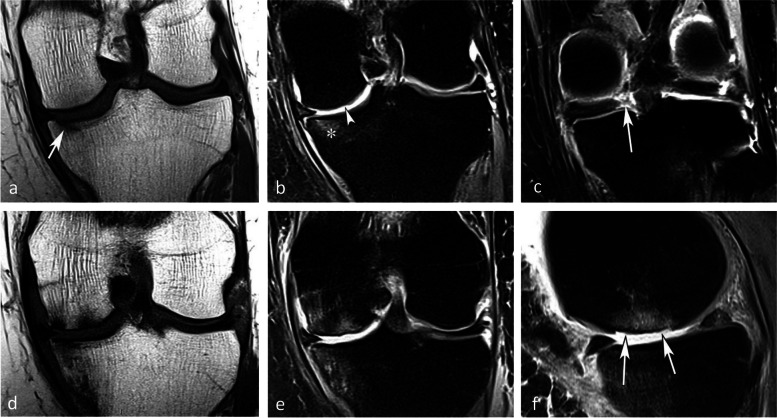
Pejorative evolution of SIF related to cartilage degradation. **a**, **b** Initial T1-weighted MR image shows a typical subchondral insufficiency fracture in the medial tibial plateau on T1-weighted image (arrow in **a**) with a BME-like high-signal intensity on fat-suppressed T2-weighted image (asterisk in **b**). **c** The lesion is associated with a radial tear of the posterior horn of the medial meniscus (arrow). **d**–**f** Six months later, T1-weighted (**d**) and fat-suppressed T2-weighted (**e**) images show various signal intensities in bone on both sides of a severe chondral loss (arrows in **f**). The initial examination already showed a clear thinning of the cartilage of the medial condyle (arrowhead in **b**), which could have indicated a pejorative evolution

It is important clinically to distinguish BME-signal changes related to osteoarthritis, from those related to SIF, because the latter may be reversible, whereas in progressive osteoarthritis, the prognosis depends on the cartilage disease rather than the BME-like signal intensity (Fig. 29). When facing a BME-like signal, the radiologist should diligently look for cartilage abnormalities. Indeed, the presence of a cartilage lesion may not always be immediately apparent and may require a thorough and detailed examination.

In brief, in the event of a BME-like pattern secondary to a cartilage lesion, the prognosis does not depend on the BME but on the evolution of the cartilage disease itself.

## Osteonecrosis of systemic origin

### Nosological context

Ischemic or avascular osteonecrosis results from impaired blood supply to the bone, causing irreversible death of bone cells (osteoblasts, osteoclasts, osteocytes) and bone marrow cells (mainly adipocytes in the knee epiphysis) [9].

Apart from traumatic causes, osteonecrosis can occur by systemic mechanisms that are not always well understood, in subjects with various risk factors (including hypercorticism, alcoholism, hyperuricemia, lupus erythematosus, sickle cell disease, and HIV) [3, 47, 65]).

**Fig. 30:**
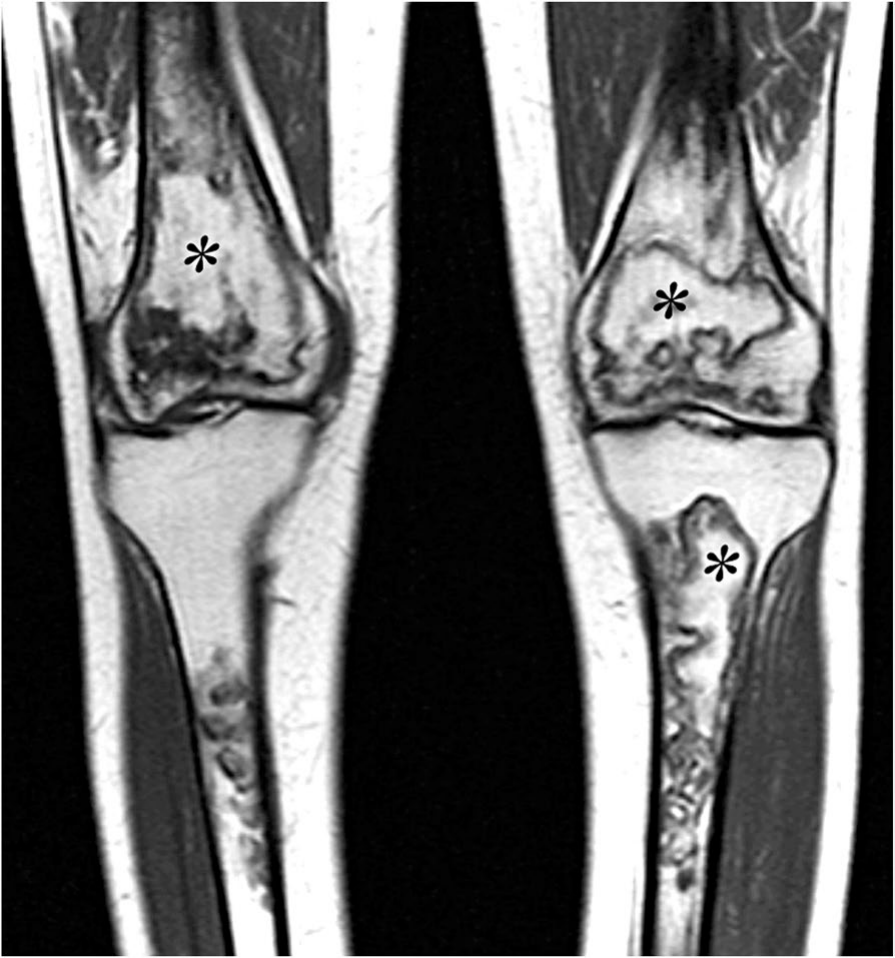
Distribution of osteonecrosis from systemic origin. T1-weighted MR image shows lesions characterized by bone marrow areas surrounded by a peripheral rim (asterisks). These lesions are often bilateral, epiphyseal, and diaphyseal-metaphyseal. The areas within the peripheral rims may show a normal marrow signal, corresponding to mummified but not degraded fat

Systemic osteonecrosis occurs in the epiphyses and in the diaphyseal and metaphyseal regions, particularly in or near the hips and knees. Lesions are bilateral in more than 80% of cases (Fig. 30) [2].

Depending on the location, osteonecrosis was previously termed “avascular necrosis,” “aseptic necrosis,” or “ischemic necrosis” (if located in the epiphysis), or “bone infarction” (if located in the metaphysis or diaphysis) [9]. However, the term osteonecrosis is appropriate for all locations of devitalized bone [65].

In the absence of mechanical collapse, these lesions are generally asymptomatic (so-called silent osteonecrosis). It is when a collapse occurs that the lesion becomes symptomatic [2, 25].

### Radiographic appearance

**Fig. 31:**
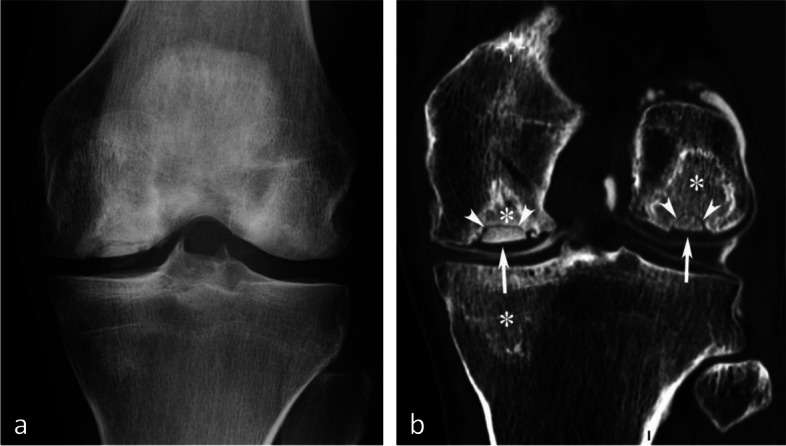
Radiographic of osteonecrosis of systemic origin. **a** Radiograph shows areas of heterogeneous bone sclerosis, in this case particularly prominent in the condyles. **b** CT arthrography shows areas of irregular bone densification corresponding to osteonecrotic lesions (asterisks) surrounded by sclerotic rims. Note collapsed segments in the weight-bearing areas of inferior condylar poles (arrows) associated with fractures within necrotic areas (arrowheads)

Radiography is normal for early lesions. In more advanced stages, osteonecrosis appears as irregular densification with a serpentine sclerotic border, better depicted on CT (Fig. 31).

The collapse of an epiphyseal lesion is associated with deformation or disruption of the subchondral bone plate, sometimes with separation of the subchondral bone plate from the rest of the lesion (i.e., subchondral dissection). Epiphyseal deformity may evolve towards osteoarthritis [65].

### MRI appearance

On MRI, osteonecrosis of systemic origin appears as an area of yellow marrow surrounded by a serpentine or curvilinear low signal intensity rim on all sequences [65]. The rim corresponds to the reactive tissue around the area of osteonecrosis. When the necrotic area extends to the bone surface, the edge of the peripheral rim presents a roughly hemispherical or conical appearance occupying part of the epiphysis [65].

**Fig. 32:**
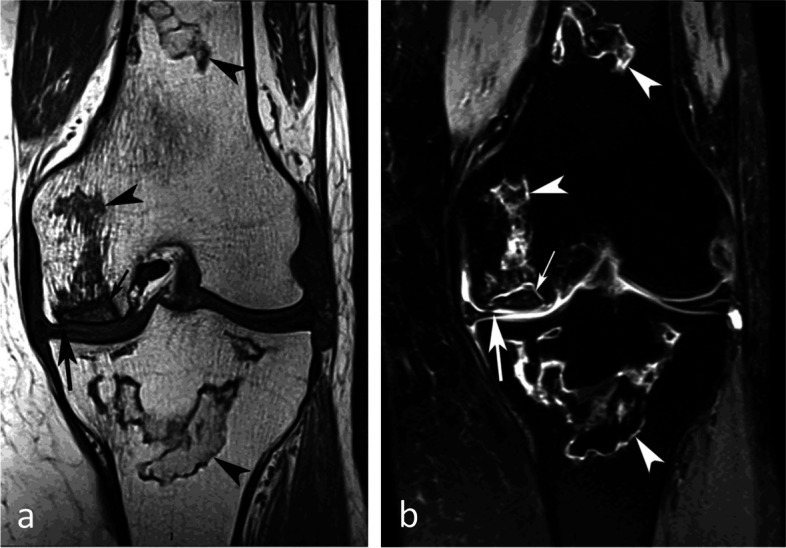
MRI pattern of osteonecrosis of systemic origin. **a**, **b** T1-weighted (**a**) and fatsuppressed T2-weighted MR images (**b**) show disseminated areas of osteonecrosis surrounded by linear rims appearing in low or high signal intensity on T1- or T2-weighted images, respectively (arrowheads). A necrotic collapsed area is shown, with fracture in the corresponding intraosseous lesion (arrows)

In non-collapsed lesions, the content of the necrotic area has a normal fatty appearance, hyperintense on T1-weighted images. It corresponds to “mummified” fatty marrow [59, 66]. In collapsed lesions, areas of modified necrotic marrow show low signal intensity on T2-weighted images, due to progressive physicochemical degradation, or saponification of the medullary fat (Fig. 32) [59]. In the acute phase of collapse, the bone marrow around areas of osteonecrosis may show BME-like signal [8, 67].

**Fig. 33:**
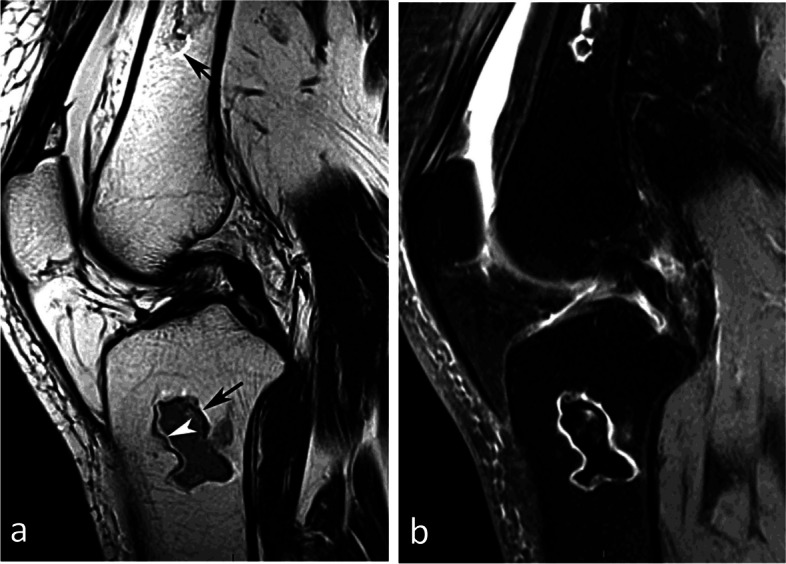
Double line rim in MRI. **a** In the illustrated case, on T2-weighted image, high signal intensity lines project behind the low signal intensity lines at the anterior aspect of the osteonecrotic areas (arrowhead), but also behind the lines with low signal at the posterior part of the osteonecrotic areas (arrows). This shifted position of the lines in the same direction in front and behind the necrotic areas is consistent with a chemical shift artifact. **b** On fat-suppressed T2-weighted image, the low signal component of the cellular rim has the same signal as the suppressed fat, and therefore a double rim line cannot be seen

The peripheral rim may show a double-line pattern on T2-weighted images. The outer, low signal intensity line is generally considered to correspond to a layer of sclerosis, and the inner, high signal intensity line to a layer of granulation tissue [65, 68]. This sign is considered pathognomonic for osteonecrosis [1, 65, 66]. However, this double line could also correspond to “chemical-shift artifact” [59, 69] (Fig. 33a). With fat suppression, the outer low-intensity line may be completely invisible, as it can be obscured by the surrounding low-intensity fat (Fig. 33b).

### Evolution and prognosis

Osteonecrosis located in the diaphyses or metaphyses has a good prognosis, just as epiphyseal lesions that do not contact the articular surface [2].

For the epiphyseal lesions contacting the articular surface, the prognosis is influenced by the dimensions of the articular surfaces concerned, as for SIF-ONs. One method of evaluation consists in measuring the angles covering the necrotic surfaces from the center of the condyles, on frontal and profile radiographs. When the combined necrotic angle is greater than 250°, the prognosis is significantly worse [2].

## Conclusions

Spontaneous epiphyseal lesions include simple insufficiency fractures (SIFs) and osteonecrosis complicating insufficiency fractures (SIF-ONs), which constitute a common nosological entity, with different prognoses. Indeed, there are two types of subchondral insufficiency fractures: those that heal (i.e., SIFs) and those that evolve poorly towards osteonecrosis (i.e., SIF-ONs) and collapse. In the early stages, analysis of the subchondral area can help recognize the risk of progression from a SIF to a SIF-ON. A collapsed articular surface evolves towards osteoarthritis, especially when large. When the collapse is very limited, it may remain stable over time.

BME-like signal may also be related to cartilage lesions and be mistaken for a sign of SIF. However, BME-like signal secondary to cartilage lesions should be recognized as such, as their prognosis does not depend on the BME-like changes but on the evolution of the cartilage disease itself.

Osteonecrosis of systemic origin results from ischemia, is delimited by a characteristic serpentine peripheral rim, and is sometimes complicated by epiphyseal collapse.

Finally, while CRPS 1 may share some similarities with SIF, it is generally characterized by a more intense BME-like signal, longer-lasting symptoms, and a tendency to migrate to different locations. Furthermore, in the vast majority of cases, CRPS 1 is still reversible.

## Data Availability

Not applicable.

## Abbreviations

BME: Bone marrow edema
CRPS: Complex regional pain syndrome
MRI: Magnetic resonance imaging
SIF-ON: Subchondral insufficiency fracture with osteonecrosis
SIF: Subchondral insufficiency fracture
SONK: Spontaneous osteonecrosis of the knee
STIR: Short-tau inversion recovery
T2FS: Fat-suppressed T2-weighted MR image (and all types of fat-suppressed fluid-sensitive images)
